# Cognitive-Enhancing Effect of Steamed and Fermented *Codonopsis lanceolata*: A Behavioral and Biochemical Study

**DOI:** 10.1155/2014/319436

**Published:** 2014-06-16

**Authors:** Jin Bae Weon, Bo-Ra Yun, Jiwoo Lee, Min Rye Eom, Hyun-Jeong Ko, Hyeon Yong Lee, Dong-Sik Park, Hee-Chul Chung, Jae Youn Chung, Choong Je Ma

**Affiliations:** ^1^Department of Medical Biomaterials Engineering, College of Biomedical Science, Kangwon National University, Hyoja-2 Dong, Chuncheon 200-701, Republic of Korea; ^2^Laboratory of Microbiology and Immunology, College of Pharmacy, Kangwon National University, Chuncheon 200-701, Republic of Korea; ^3^Department of Teaics, Seowon University, Cheongju 361-742, Republic of Korea; ^4^Functional Food & Nutrition Division, Department of Agrofood Resources, Rural Development Administration, Suwon 441-853, Republic of Korea; ^5^Newtree Co., Ltd., 11F Tech Center, SKnTechno Park 190-1, Sungnam 462-120, Republic of Korea; ^6^Research Institute of Biotechnology, Kangwon National University, Chuncheon 200-701, Republic of Korea

## Abstract

Alzheimer's disease (AD) is a progressive neurodegenerative disorder characterized by memory impairment. *Codonopsis lanceolata* (*C. lanceolata*) has been employed clinically for lung inflammatory diseases such as asthma, tonsillitis, and pharyngitis. The present study was undertaken to evaluate the effect of fermented *C. lanceolata* (300, 500, and 800 mg/kg) on learning and memory impairment induced by scopolamine by using the Morris water maze and passive avoidance tests. To elucidate possible mechanism of cognitive-enhancing activity, we measured acetylcholinesterase (AchE) activity, brain-derived neurotrophic factor (BDNF), and cyclic AMP response element-binding protein (CREB) expression in the brain of mice. Administration of fermented *C. lanceolata* (800 mg/kg) led to reduced scopolamine-induced memory impairment in the Morris water maze and passive avoidance tests. Accordingly, the administration of fermented *C. lanceolata* inhibited AchE activity. Interestingly, the level of CREB phosphorylation and BDNF expression in hippocampal tissue of scopolamine-treated mice was significantly increased by the administration of fermented *C. lanceolata*. These results indicate that fermented *C. lanceolata* can ameliorate scopolamine-induced memory deficits in mouse and may be an alternative agent for the treatment of AD.

## 1. Introduction

Alzheimer's disease (AD) is a progressive neurodegenerative disorder that results in impaired memory and cognition and is the most common cause of dementia among older people [1, 2]. There are multiple causes of AD and some causes have yet to be discovered. The pathogenesis of AD is defined by the presence of senile plaques, neurofibrillary tangles, and several biochemical factors such as inflammation and oxidative stress. Plaques are formed by the accumulation of *β*-amyloid, and inflammation around plaques in the brain can lead to cell death. Neurofibrillary tangles consist of the protein tau, a microtubule-associated protein. The presence of neurofibrillary tangles, together with the accumulation of *β*-amyloid, interferes with normal cellular functioning.

Acetylcholine (Ach), a neurotransmitter of the cholinergic system, plays an important role in memory and information processing. Decreased levels of Ach as well as the Ach synthesizing enzyme choline acetyltransferase (CHAT) in the cerebral cortex are another neuropathology associated with AD [3]. Acetylcholinesterase (AChE) inhibitors, including donepezil, galantamine, and tacrine, and an Ach receptor agonist have been used in the treatment of AD [4, 5]. However, these medicines have several side effects, such as pain, nausea, and vomiting. Medicinal plants such as* Ginkgo biloba*,* Salvia officinalis* (sage),* Melissa officinalis* (balm), and* Papaver somniferum* (opium poppy) are reported to have beneficial effects in patients with AD and to have fewer side effects [6]. Scopolamine, a muscarinic cholinergic antagonist, has been used to develop animal models of AD and can be used to study the effects of antiamnesic drugs [7]. Scopolamine increases AchE activity in the cortex and hippocampus. In addition, it impairs mitochondrial function and reduces ATP levels [8].

Brain-derived neurotrophic factor (BDNF) plays a role in learning and memory formation and is regulated via transcription factor and cAMP-response element-binding protein (CREB). Downregulation of BDNF expression is associated with memory impairment [9–11].

*Codonopsis lanceolata *(Campanulaceae) is a traditional medicinal plant used for the treatment of hypertension and several lung inflammatory diseases, such as asthma, tonsillitis, and pharyngitis; it has been used in East Asia for thousands of years [12, 13].* C. lanceolata* contains various compounds such as saponins, alkaloids, tannins, steroids, and polysaccharides [14]. Previous reports have shown that* C. lanceolata* inhibits the production of TNF-*α* and nitric oxide, the expression of interleukin (IL)-3 and IL-6, and LPS-mediated phagocytic uptake in RAW 264.7 cells (regulatory effects of* C*.* lanceolata* on macrophage-mediated immune responses) [15]. It also shows antilipogenic and anti-inflammatory effects in mice with alcohol-induced fatty liver [16]. Lancemaside A, major compounds in* C. lanceolata,* showed enhancing effect of memory, inhibiting AChE activity, and inducing BDNF and p-CREB expressions [17].

Recently, we verified that the fermented* C. lanceolata* ameliorated memory and learning impairment and the steamed and fermentation process significantly improved the cognitive recognition activity compared to original* C. lanceolata *[18–20]. In the present study, we modified the steamed and fermentation process for mass production of fermented* C. lanceolata* and confirmed the effect of fermented* C. lanceolata* on memory and learning impairment using scopolamine-induced memory deficits in the Morris water maze and passive avoidance tests. We investigated the possible mechanism of cognitive enhancement effect of fermented* C. lanceolata* and found that the administration of fermented* C. lanceolata *inhibited AchE activity. Intriguingly, the administration of fermented* C. lanceolata* also increased the expression of BDNF in accordance with increased CREB phosphorylation in hippocampus. Collectively, these results suggest that fermented and steamed* C. lanceolata *can ameliorate scopolamine-induced memory deficits in mouse and may be a possible agent for the treatment of AD.

## 2. Materials and Methods

### 2.1. Plant Materials

The roots of* C. lanceolata* were collected from Hoengseong, Gangwon, Korea. The* C. lanceolata* was washed several times with tap water to remove foreign material and dried in the shade at 20–30°C for 2 days. Dried* C. lanceolata* was steamed using a steam device (Dechang Stainless, Seoul, Korea) at 90°C for 8 h and this steaming process was repeated 5 times.

### 2.2. Fermentation and Extraction

*Bifidobacterium longum* (KACC 20587),* Lactobacillus acidophilus* (KACC 12419), and* Leuconostoc mesenteroides* (KACC 12312) were obtained from the Korean Agricultural Culture Collection (Suwon, Korea). The steamed* C. lanceolata* was mixed in distilled water 8 times the weight of the* C. lanceolata* and aseptically inoculated with approximately 10^6^ CFU/g of* Bifidobacterium longum*,* Lactobacillus acidophilus*, and* Leuconostoc mesenteroides *(1 : 1 : 1). The inoculated* C. lanceolata* was fermented for 48 h at 30°C. Then, fermented* C. lanceolata* was extracted in 70% ethanol with 5 times the weight of the* C. lanceolata* at 80°C. This extract was concentrated using 24 h of reflux extraction at 70°C and harvested by centrifuging at 15,000 rpm. After evaporation, spray drying was conducted to obtain the fermented* C. lanceolata*.

### 2.3. Animals

ICR mice (3-week-old males; weight: 25–30 g) were obtained from the Dae Han Biolink Co. (Eumseong, Korea). The mice had access to commercial pellet feed and water* ad libitum*. Mice were housed 7 per cage and kept in a temperature-controlled room (20 ± 3°C) with a 12/12-h light-dark cycle. They were used after a 1-week adaptation period. All animal experiments in this study were carried out in accordance with the guidelines of the Kangwon National University IACUC (KIACUC).

### 2.4. Scopolamine Injection and Drug Administration

In Morris water maze test, the mice were administered 0.5% carboxymethylcellulose (CMC; control group), fermented* C. lanceolata* (300, 500, and 800 mg/kg, dissolved in CMC), and donepezil (used as a positive control; 1 mg/kg) orally 90 min before treatment with scopolamine. The control group received normal saline, subcutaneously (SC), and all other groups were given scopolamine (1 mg/kg SC, dissolved in saline) to induce amnesia. The first trial of the test was performed 30 min after scopolamine treatment.

In passive avoidance test, the mice were administered 0.5% CMC (control group), fermented* C. lanceolata* (300, 500, and 800 mg/kg), and donepezil (1 mg/kg) orally 90 min before treatment with scopolamine. Scopolamine (1 mg/kg SC, dissolved in saline) was administered 30 min prior to testing.

### 2.5. Morris Water Maze Test

The water maze test was performed as described in our previous study [18, 21]. The water maze consisted of a circular pool (90 cm diameter and 40 cm height) filled with water up to 30 cm and maintained at 20 ± 1°C and areas of the maze were defined as four equal quadrants. Starting points on the outside of the pool were changed each day. A white escape platform (10 cm diameter and 26 cm height) was placed in the center of one quadrant and submerged 1 cm below the surface of the water. All swimming behaviors of the mice were monitored and analyzed by Smart (ver. 2.5.21) video-tracking system. The escape latency and the time to locate the platform were used as measures for the development of spatial memory. Mice were given 60 s to swim in the absence of the platform on the day of the probe trial. The mice received two trial sessions per day for 4 consecutive days, with a 20 min intertrial interval. Location of the platform was unchanged between trial 1 and trial 2 during the test period. For the probe trial, the platform was removed for a period of 60 s on the last day. The time spent in the target quadrant was investigated to determine the memory of the mice.

### 2.6. Passive Avoidance Test

The passive avoidance test was carried out as previously described [18]. The mice were tested in a passive avoidance apparatus (Gemini, San Francisco, USA), which consisted of two equally sized compartments (17 cm × 12 cm × 10 cm) with an electrifiable grid floor. The two compartments were divided by a guillotine door. On the first day, the mice performed a training trial in the avoidance apparatus. Twenty-four hours after the training trial, the mice were initially placed in the light compartment and after 20 s the door between the two compartments was opened. Movement of the mice in the dark compartment caused the guillotine door to close. An electric footshock (0.1 mA/10 g body weight, 2 s duration) was then delivered through the grid floor. During each trial, the time taken to move to the dark compartment was recorded as the latency time. The mice were again placed in the light compartment and the latency time was measured 24 h later. The maximum entry latency time to darkroom was 180 s.

### 2.7. Acetylcholinesterase Activity Determination

Acetylcholinesterase (AChE) activity test described by Ellman method was used with slight modification [22]. Mice were euthanized after behavioral test, and brain was removed. Hippocampus was dissected out from the brain and rapidly homogenized with sodium phosphate buffer. The mixture contained 33 *μ*L of homogenate, 470 *μ*L of sodium phosphate buffer, and 167 *μ*L of DTNB. Then, 280 *μ*L of acetylcholine iodide was added to the reaction mixture. After incubation, the reaction was measured at 412 nm using spectrophotometer. The AChE activity was calculated as the optical density value per mg protein.

### 2.8. Tissue Preparation and Western Blot Analysis

Mice were sacrificed 30 min after last behavior test. The brains were promptly collected and hippocampus was excised. Hippocampal tissues were homogenized in 200 *μ*L of ice-cold RIPA buffer containing a protease inhibitor cocktail and centrifuged at 13,000 ×g for 20 min. The supernatants were stored at −80°C. The supernatant containing 20 *μ*g of protein was subjected to 15% SDS-PAGE for 2-3 h at 100 V and transferred to PVDF membrane. The membrane was blocked in 5% skim milk for 1 h at room temperature and incubated overnight with a 1 : 2000 dilution of *β*-actin, 1 : 1000 dilution of CREB, 1 : 500 dilution of pCREB and 1 : 1000 dilution of BDNF antibody at 4°C. After incubation, the membrane was washed three times with 0.1% PBST.

Secondary antibodies (goat-anti-rabbit IgG HRP 1 : 2000 for BDNF, donkey-anti-goat IgG HRP 1 : 2000 for pCREB, and goat-anti-mouse IgG HRP 1 : 2000 for CREB and *β*-actin) were conjugated for 2 h at room temperature and then were washed three times with 0.1% PBST.

### 2.9. Statistical Analyses

Data from the Morris water maze test were analyzed by two-way ANOVA. Data from the probe trial test, passive avoidance test, and AChE activity values were analyzed by one-way ANOVA. All results were expressed as means ± SEM. If the results were significant, significant differences in direct comparisons were determined by Tukey's post hoc test. Statistical significance was set at *P* < 0.05, *P* < 0.01, and *P* < 0.001.

## 3. Results

### 3.1. Morris Water Maze Test

**Figure 1:**
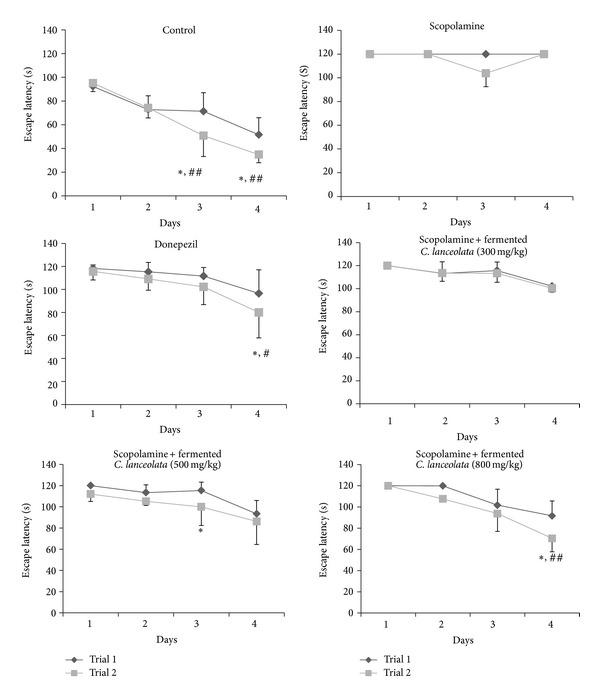
The effect of fermented* C. lanceolata* on escape latency in scopolamine-injected mice in the Morris water maze test. Control group (0.5% CMC (10 mL/kg body weight, P.O.) scopolamine group (1 mg/kg body weight, S.C.), donepezil group (1 mg/kg body weight, P.O.)) and fermented* C. lanceolata* group (300, 500, and 800 mg/kg body weight, P.O. treated 1 h before scopolamine administration). The values shown are the mean escape latency ± SD (*n* = 7). Escape latency of trial 2 significantly differed from that of trial 1: *P < 0.05, **P < 0.01, and ***P < 0.001 and ^#^ *P* < 0.05, ^##^ *P* < 0.01, and ^###^ *P* < 0.001 versus the scopolamine group.

Based on our recent report, we found that the steamed and fermented* C. lanceolata* showed significantly increased cognitive enhancement activity. After modifying the steam and fermentation process for the mass production of steamed and fermented* C. lanceolata,* we investigated the effect of newly processed steamed and fermented* C. lanceolata* extract on scopolamine-induced spatial memory impairment by using the Morris water maze test (Figure 1).

**Figure 2:**
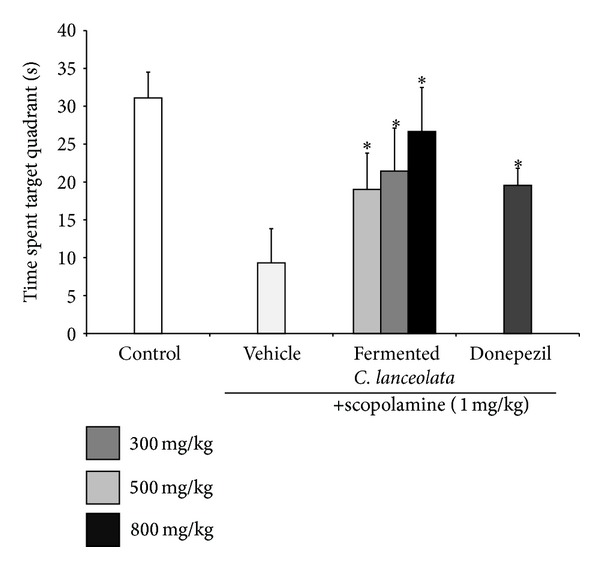
Mean escape latency of each group in the probe trial. The time spent in the quadrant where the platform was previously placed. Data represent the mean ± SD. *P < 0.05, **P < 0.01, and ***P < 0.001 versus the scopolamine group.

**Figure 3:**
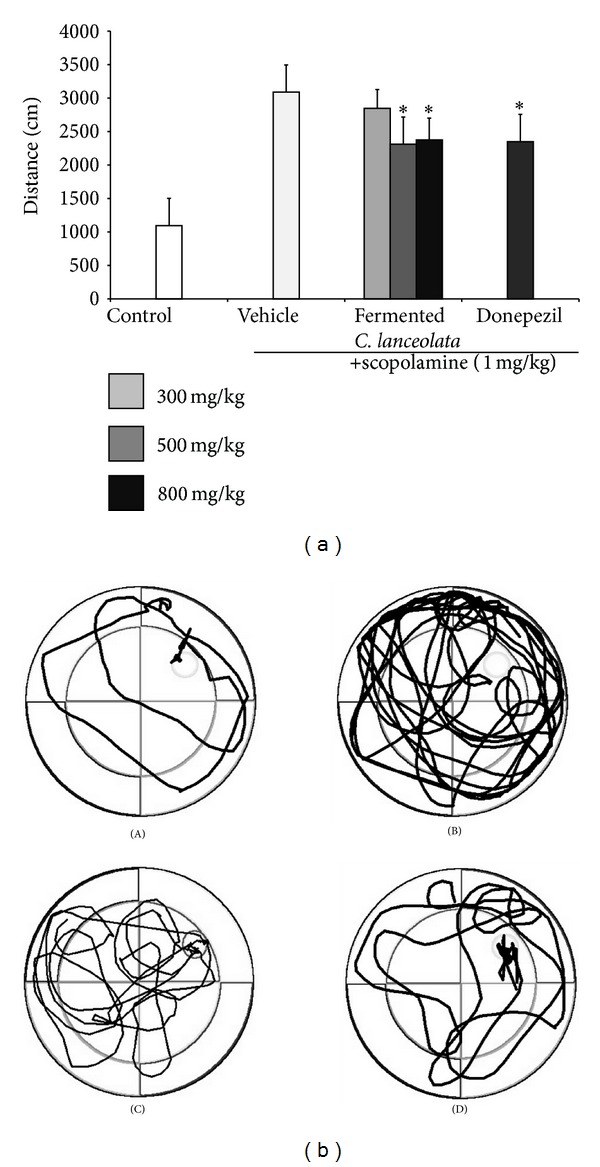
(a) Mean distance travelled to the platform in the Morris water maze test. Results are expressed as mean ± SD (*n* = 7). *P < 0.05, **P < 0.01, and ***P < 0.001 compared with scopolamine group. (b) Typical swimming routes of each group in the Morris water maze test. (A) Control group; (B) scopolamine-treated group; (C) scopolamine + donepezil-treated group; (D) scopolamine + fermented* C. lanceolata* (800 mg/kg, P.O.).

The scopolamine-treated group did not show decreased escape latency time during the 4 trial days. The steamed and fermented* C. lanceolata* group showed shorter escape latency than the scopolamine-treated group from trial 1 and trial 2 after the 1st trial day. The donepezil-treated group also showed significantly reduced escape latency from trial 2 during the 3rd and 4th trial day. In the Morris water maze test, significant effects were observed for the fermented* C. lanceolata* group for treatment [*F*(6, 98) = 2.21, *P* < 0.001], for days [*F*(3, 98) = 2.87, *P* < 0.001], and for the interaction between treatment and day [*F*(18, 98) = 1.82, *P* = 0.95]. Compared to the control group, the scopolamine group spent less swimming time in the target quadrant after the platform was removed following the probe trial on the last day. The fermented* C. lanceolata* group showed significantly increased swimming time in the target quadrant than did the scopolamine group (Figure 2). Fermented* C. lanceolata* (800 mg/kg, P.O.) significantly decreased the scopolamine-induced increase in the average distance to the platform during the water maze test. The typical swimming routes of each group are shown in Figure 3.

### 3.2. Passive Avoidance Test

**Figure 4:**
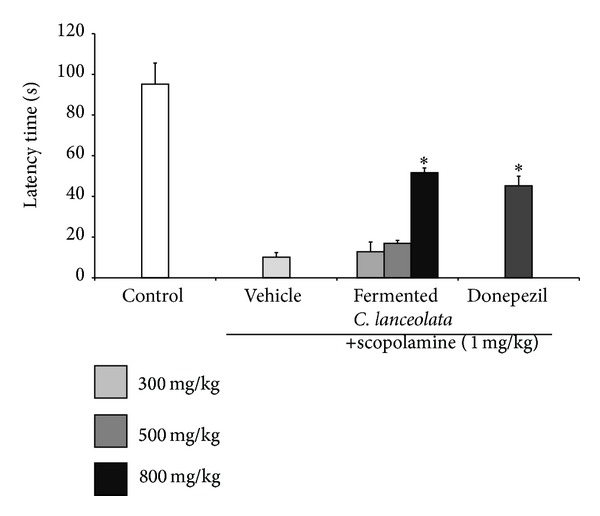
Effect of fermented* C. lanceolata* on scopolamine-induced memory impairment in the passive avoidance test. Mean latency time (s) ± SD (*n* = 7). *P < 0.05, **P < 0.01, and ***P < 0.001 compared with the scopolamine group.

We also investigated the effect of newly processed steamed and fermented* C. lanceolata* on scopolamine-induced memory deficit in the passive avoidance test. Passive avoidance test is generally used to assess long-term memory mice [23]. The latency time in the scopolamine group was significantly decreased as compared to that in the control group. Compared to the scopolamine-treated mice, the fermented* C. lanceolata*-treated mice (800 mg/kg, P.O.) that were cotreated with scopolamine exhibited significant reversal of latency time. This effect increased in a dose-dependent manner (Figure 4). Donepezil, an acetylcholinesterase inhibitor, was used as positive control. Treatment with donepezil also showed a significant increase in latency time. In this study, the steamed and fermented* C. lanceolata*-treated group (800 mg/kg, P.O.) showed similar latency time to donepezil-treated group.

### 3.3. Acetylcholinesterase Activity Determination

Since AchE activity was reported to be involved in memory impairment, the effect of fermented* C. lanceolata* on AchE activity in hippocampus of mice was evaluated, as shown in Table 1. Scopolamine treatment significantly increased AchE activity in hippocampus as compared with those of control group. Fermented* C. lanceolata* (800 mg/kg) treatment significantly decreased AchE activity by 35.63%. After treatment with donepezil, AchE activity of scopolamine-treated mice was decreased by 27.59%. This result demonstrated that the steamed and fermented* C. lanceolata* exerts cognitive-enhancing effect through the inhibition of AchE in hippocampus.

### 3.4. The Effect of Steamed and Fermented* C. lanceolata* on BDNF Expression and CREB Phosphorylation

The activation of CREB and BDNF plays a role in the enhancement of memory. Furthermore, increased CREB and BDNF activation improved long-term memory [24]. Further, scopolamine treatment was known to be involved in the inhibition of BDNF expression and CREB phosphorylation (pCREB). Thus, we decided to assess whether the effect of steamed and fermented* C. lanceolata* ameliorated memory impairment was linked to the increased BDNF and CREB activation in the hippocampus of mice. To determine the effect of steamed and fermented* C. lanceolata* on the BDNF and pCREB expression reduced by scopolamine in hippocampus, we conducted western blot analysis.

**Figure 5:**
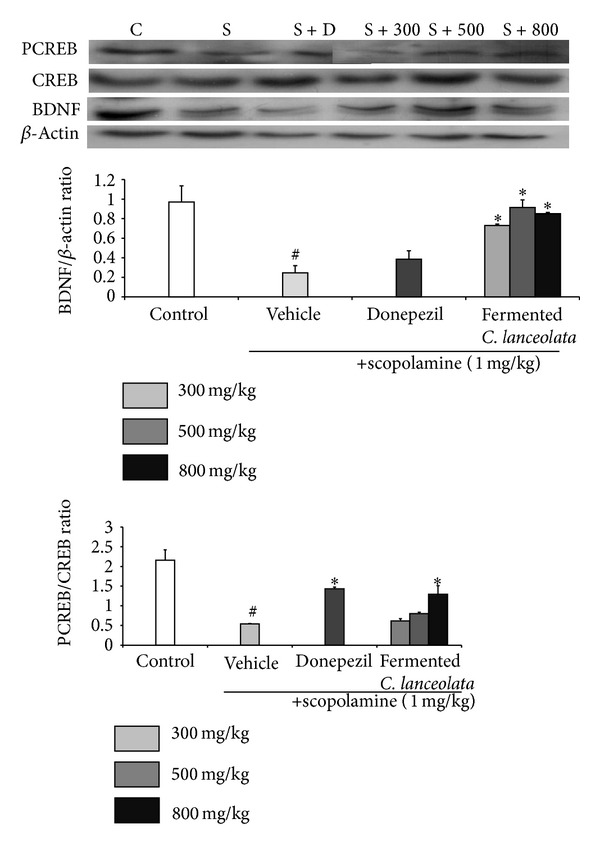
The proton levels of BDNF and pCREB in hippocampus. Control group (C; 0.5% CMC (10 mL/kg body weight, P.O.)), scopolamine group (S; 1 mg/kg body weight, S.C.), donepezil group (S + D; 1 mg/kg body weight, P.O.), and fermented* C. lanceolata* group (S + 300, S + 500, and S + 800; 300, 500, and 800 mg/kg body weight, P.O. treated 1 h before scopolamine administration) (*n* = 5 per group; ^#^ *P* < 0.05, ^##^ *P* < 0.01, and ^###^ *P* < 0.001 versus the control group; *P < 0.05, **P < 0.01, and ***P < 0.001 versus the scopolamine group).

As shown in Figure 5, the BDNF expression in hippocampus was decreased following exposure of scopolamine to mice. The BDNF expression in mice with the steamed and fermented* C. lanceolata* (300, 500, and 800 mg/kg, P.O.) administration is significantly increased compared to scopolamine-treated mice. Scopolamine administration also resulted in a reduced pCREB expression compared to the control group. BDNF level was significantly elevated in mice treated with fermented* C. lanceolata* (800 mg/kg, P.O.) compared to scopolamine-treated mice. A correlation was observed between results in behavioral tests and levels of pCREB and BDNF.

## 4. Discussion

We established a new steamed and fermented method for large-scale production of fermented* C. lanceolata*. This method can be used with commercial application of fermented* C. lanceolata*.

The present study evaluated the cognitive-enhancing effect of fermented* C. lanceolata* extract by modified process on scopolamine-induced memory impairment in mice. The Morris water maze test is one of the most commonly used tests to investigate hippocampal-dependent spatial learning and memory in mice. The passive avoidance test is also used to assess memory retention based on the natural tendency of animals formed by an aversive stimulus. Scopolamine, a competitive muscarinic acetylcholine receptor antagonist, inhibits cholinergic activity. Cholinergic deficiency is associated with the cognitive impairments observed in AD [25, 26]. We found that fermented* C. lanceolata* extract improved memory and learning in mice with scopolamine-induced deficits in the Morris water maze and passive avoidance tests. In the Morris water maze test, the fermented* C. lanceolata- *(800 mg/kg) treated mice exhibited significantly shorter escape latency on day 4, between the 1st trial and 2nd trial, suggesting an improvement in short-term memory. The memory deficits induced by scopolamine were reversed significantly by treatment with fermented* C. lanceolata* extract at a dose of 800 mg/kg. These results implied that new large-scale process has stability of memory-enhancing activity of fermented* C. lanceolata*.

AchE is the enzyme involved in Ach hydrolysis at central cholinergic synapses. AchE activity in hippocampus was increased by administered scopolamine [27]. This study showed fermented* C. lanceolata* extract inhibited AchE activity in a doses-dependent manner. The hippocampal neurogenesis is important for learning and memory. Increased neurogenesis improved memory and new neurons increase memory capacity. Regulation of gene expression via BDNF plays a role in the long-term potentiation (LTP) and memory formation [28]. BDNF belongs to the neurotrophin family and activity is corrected with increased neurogenesis in the dentate gyrus of the hippocampus and enhanced memory [29]. Elevation of CREB activity leads to an upregulation of BDNF [30]. CREB promotes transcription of many specific target genes, including BDNF by phosphorylation at serine 133. CREB-mediated transcription interacts with coactivator CREB-binding protein [31, 32]. We tested the effect of fermented* C. lanceolata* on CREB phosphorylation (pCREB) and BDNF expression. Scopolamine reduced pCREB and BDNF levels in the hippocampus. We observed that fermented* C. lanceolata* treatment increased BDNF and pCREB levels.

Fermented* C. lanceolata* improved scopolamine-induced memory deficit during behavioral tests. In addition, fermented* C. lanceolata* inhibited AchE activity and increased pCREB and BDNF expression. These results of the present study suggest that the effect of fermented* C. lanceolata* may be associated with the muscarinic cholinergic receptor and may reverse cognitive impairment by affecting brain cholinergic activity. Moreover, the memory improving activity of fermented* C. lanceolata* is exerted via upregulation of pCREB and BDNF in hippocampus. Our results support new mechanism of cognitive-enhancing effect of fermented* C. lanceolata* through BDNF and pCREB expression.

In summary, fermented* C. lanceolata* exhibited a cognitive-enhancing effect in the Morris water maze and passive avoidance test by inhibition of AchE activity and elevation of BDNF and pCREB expression. Further studies are needed to determine the role of fermented* C. lanceolata* in CREB and BDNF expression pathway.

## Figures and Tables

**Table 1:** Acetylcholinesterase inhibitory effects of fermented *C. lanceolata* on memory deficit in mice.

Groups	AchE activity (U/mg protein)
Control	0.68 ± 0.038
Scopolamine	0.87 ± 0.008
Donepezil	0.63 ± 0.023
Fermented *C. lanceolata *	
300 mg/kg	0.74 ± 0.043
500 mg/kg	0.63 ± 0.048
800 mg/kg	0.56 ± 0.021***

Data represent the mean ± SD. ***P < 0.001 versus the scopolamine group.
